# Comparison between canine and porcine models of chronic deep venous thrombosis

**DOI:** 10.1186/s12959-023-00565-5

**Published:** 2023-12-06

**Authors:** Chuang Wang, Tao Tang, Sheng-Lin Ye, Nan Hu, Xiao-Long Du, Xiao-Qiang Li

**Affiliations:** Department of Vascular Surgery, Nanjing Drum Tower Hospital, Affiliated Hospital of Medical School, Nanjing University, #321 Zhongshan Road, Nanjing, 210008 Jiangsu China

**Keywords:** Venous thrombosis, Animal model, Vascular patency

## Abstract

**Objective:**

To first induce chronic deep venous thrombosis in the left iliac veins of canines and porcines and then compare these two models to validate endovascular treatment devices.

**Methods:**

Thrombin and fibrinogen were used to produce a solid thrombus in the left iliac veins of a stenosis model. The researchers used venous angiography and histological staining to investigate the progression of thrombosis.

**Results:**

A left iliac vein thrombus was successfully formed in all experimental animals, including six Labrador dogs and three Bama miniature pigs, and there was minimal surgical bleeding. All dogs survived until 90 days, and three pigs died on Days 29, 33, and 58.

**Conclusion:**

The researchers first established the models and then observed the progression of chronic deep venous thrombosis of the iliac vein in large animals for up to 90 days. Dogs are better suited for chronic deep venous thrombosis models due to their uncomplicated anatomy, excellent obedience, and proneness to physical activity compared with pigs.

**Supplementary Information:**

The online version contains supplementary material available at 10.1186/s12959-023-00565-5.

## Introduction

Venous thromboembolism (VTE) refers to deep venous thrombosis (DVT) and pulmonary embolism, which is a worldwide problem and has very high morbidity and mortality rates. Approximately two-thirds of patients with VTE were diagnosed with DVT [1]. Research on venous thrombosis involves pathogenesis, pathophysiology, and therapeutic interventions. The use of animal models to construct venous thrombosis is an essential means to study the occurrence, development, and treatment of thrombosis. In most instances, small rodent models of acute or subacute venous thrombosis are used [2–4]. There are three main drawbacks regarding this type of study. First, in terms of the progression of DVT, chronic thrombosis affects patients’ quality of life and treatment intervention, which can lead to post-thrombotic syndrome, including venous ulcers and limb swelling [5]. Basic research on long-term chronic venous thrombosis is scarce. Second, small rodents have a very different body size and haemodynamics than humans, which play a key role in flow-restricted, venous stasis-dominated thrombosis [6]. Finally, all the new medications, interventions and devices, such as anticoagulant drugs, thrombolysis, thrombectomy devices, and endovascular treatment devices, cannot be studied with small animals.The choices of experimental animal models have changed dramatically over the past 20 years, from small murine models to large ones. Larger animal models offer more options and are more consistent with human physiology. There have been some attempts to develop large animal models of venous thrombosis. The methods of these experiments are different, but the theories are inseparable from Virchow’s triad, including vessel wall injury, blood hypercoagulable state, and haemodynamic abnormalities. The surgical approaches used included open surgical procedures, endovascular techniques, and laparoscopic techniques [7–9]. Kang et al. mentioned that 80% proximal stenosis and induced preoperative hypercoagulability could rapidly induce thrombosis in pigs and was highly consistent with the histological morphology inside the human body [10]. This differs from small animal models of thrombosis, in which simple proximal ligation of the inferior vena cava can be used for small rodent thrombosis models, thus rapidly forming a venous thrombus, whereas simple proximal ligation of the vein in large animals makes it difficult to form a thrombus, probably due to the enormous blood flow and abundant collateral compensation in large animals [11]. We think that feasible large animal models can better translate the results of therapeutic research into clinical application.

However, thus far, there has been no long-term observation of a large animal model of chronic venous thrombosis, which requires at least 3 months, and that is consistent with chronic thrombotic obstruction of the iliofemoral vein in humans. To this end, we designed a Labrador and Bama miniature pig model of left iliac venous thrombus. Because of the vascular anatomy, blood properties, and vascular physiology similar to humans, Labrador dogs and Bama miniature pigs were considered to be suitable experimental subjects. The present study reports the methods and follow-up results of this technique and the histological characteristics of the thrombus.

## Materials and methods

### Animals

All adult Labrador dogs and Bama miniature pigs (provided by Taizhou Meifengli Animal Experimental Center, Jiangsu Province) weighed 20–30 kg. There were 3 male and 3 female dogs, and 2 male pigs and one female pig. All animal experiments were performed in accordance with the Guidelines for Ethical Review of Laboratory Welfare and followed the Regulations on the Administration of Laboratory Animals of the Ministry of Science and Technology, PRC. All animals were housed in single rooms at a temperature of 16–26 ℃ and fed standardized food with free access to food and water.

### Anaesthesia

All animals received an intramuscular injection of Zoletil (7–25 mg/kg) for sedation and anaesthesia, which were maintained by 1–6% of isoflurane via endotracheal intubation during the operation. Blood pressure, heart rate, electrocardiogram, and oxygen saturation were monitored during surgery.

### Induction of venous thrombosis in the left iliac veins

**Fig. 1:**
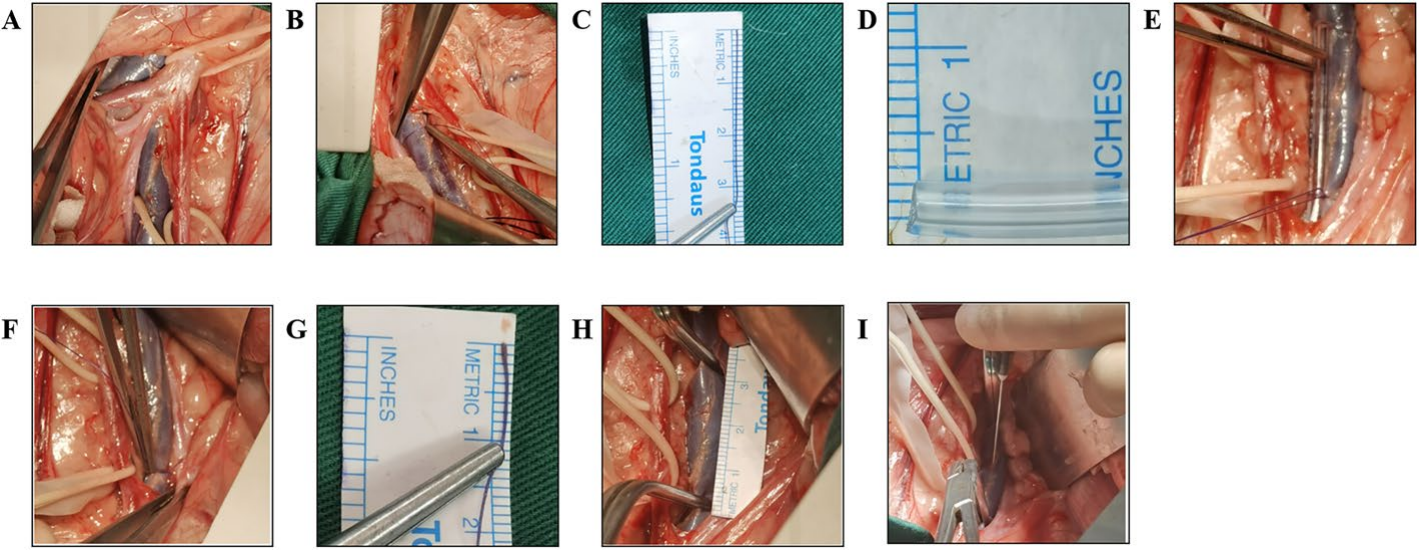
Induced venous thrombosis in the left iliac veins of canines. **A** Separated bilateral iliac veins. **B**, **C** Measured iliac vein circumference. **D** Diameter of the ligation rod. **E** Ligation of the rod and left iliac vein. **F**, **G** Remeasured iliac vein circumference. **H** Thrombosis length. **I** Injection with thrombin and fibrinogen

For this model, we established a stenosis model with thrombin, forming an immediate and solid thrombus in the left iliac vein. Animals were placed in a supine position after anaesthesia in a hybrid operating room. Conventional skin sterilization was performed in abdominal surgery, and a median abdominal incision was made from the first to the third nipple. Then, we incised the skin and subcutaneous tissues to the rectus abdominis muscle and separated the lateral peritoneum from the left abdominal wall to the posterior peritoneum. We suspended the left iliac vein and artery with an absorbable thread after exposing the abdominal aorta, inferior vena cava, and left common iliac artery and vein (Fig. 1A). The perimeter of the left common iliac vein was measured, and the diameter of the common iliac vein was calculated (Fig. 1B, C). The diameter of the ligating glass rod was calculated according to the degree of 80% stenosis (Fig. 1D). The left common iliac vein near the bifurcation of the inferior vena cava was tied with a rod of the calculated diameter, and then the rod was pulled out to show the constriction of the iliac vein at the ligation site (Fig. 1E). The diameter of the iliac vein after ligation was remeasured with silk thread, and the percentage by which the vein diameter was reduced was calculated (Fig. 1F, G). Then, the first vascular clip was placed at the ligation site, and the second clip was used at the distal end from the ligation site, taking care to avoid the branch veins. The filling and dilatation of the common iliac vein were observed, and we measured the distance between the two clips to determine the thrombus length (Fig. 1H). Immediately after that, we sequentially injected 1 ml thrombin (500 IU/mL, Harbin HanBang Medical Science and Technology Co.) and 1 ml fibrinogen (60 mg/mL, Harbin HanBang Medical Science and Technology Co.) with a 5 ml syringe under direct vision, and the blood flow was blocked with clips for 10 min (Fig. 1I). Streptomycin 300 mg and ceftiofur sodium 300 mg were given postoperatively for 7 days. Studies have shown that rivaroxaban, at a dosage of 1–2 mg/kg per day, has a significant anticoagulant effect in both pigs and dogs [12–15]. To maintain the thrombus until the chronic phase, each animal was given oral treatment with rivaroxaban tablets at 20 mg per day, starting 2 weeks after thrombosis model establishment.

### Haematological testing

Venous blood samples were collected from the right jugular vein before the induction surgery for venous thrombosis and on Days 7, 30, 60, and 90 after the surgery. Blood samples were taken from dogs and pigs in the fasting state for the following tests: prothrombin time (PT), international normalized ratio (INR), white blood cell count, neutrophil count, lymphocyte count, monocyte count, total protein, albumin, and creatinine. Testing was performed at Taizhou Meifengli Animal Experimental Center.

### Follow-up venous angiography

Left iliac vein blood flow was observed by anterograde angiography at 7, 30, 60, and 90 days after thrombus model establishment. Under anaesthesia as described previously, the left inguinal area was disinfected and sheeted. The femoral vein was located by ultrasound, a 5F puncture sheath was placed into the femoral vein, and 5 ml heparin water (8 U/mL, Shanghai ShangPharma First Biochemical Company) was injected through the catheter to prevent thrombosis from occluding the contrast catheter. Iodixanol contrast agent (100 mL, General Electric Pharmaceutical Co.) and heparin water (10 ml, 1:1) were added for imaging. The left iliac vein should be visualized, and the contrast agent should be returned to the inferior vena cava directly through the partially recanalized veins or the compensatory collateral vessels until the contrast agent dissipates in the visual field.

### Histological staining

At 90 days after venous thrombosis induction, the bilateral iliac veins of the surviving animals were isolated and fixed in 4% paraformaldehyde for at least 24 h after dissection, followed by dehydration in ethanol with a gradient of concentrations. The dehydrated samples were embedded in paraffin and serially sectioned at 4 μm thickness. These sections were subjected to haematoxylin–eosin (HE) and Masson staining and photographed by light microscopy.

### Statistical analysis

We calculated the number (%) for categorical variables and the mean ± standard deviation for normally distributed data. The results of thrombosis modelling in pigs and dogs were tested by independent sample T test (continuous variables) or Chi-square test (categorical variables). A two-sided *p* value < 0.05 represented statistically significant differences. We analyzed all data using the SPSS 26.0 statistical package and Prism 8.4.2 software.

## Results

### The canine model of chronic deep venous thrombosis

**Fig. 2:**
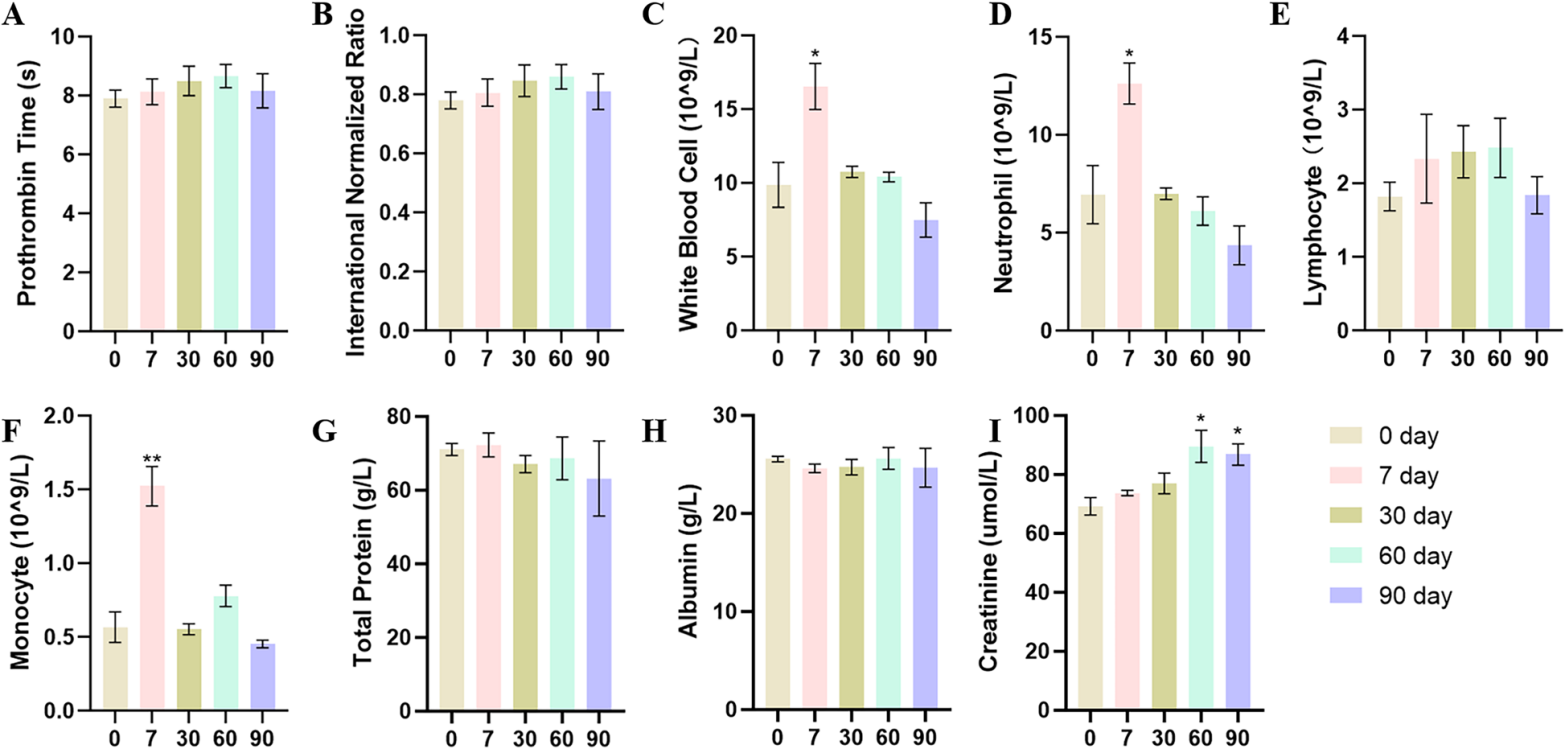
Haematological testing of dogs. * *P* < 0.05, ** *P* < 0.01, *** *P* < 0.001, all groups vs. 0 day

The underlying conditions of the animals and operative data are shown in Table 1. The perimeter of the dogs' left iliac vein was 32.0 ± 3.3 mm, and the diameter was 10.2 ± 1.1 mm. Remeasurement of the left iliac veins after ligation with a 0.2 cm diameter glass rod showed that the constricted vein diameter was 4.1 ± 0.8 mm, and the stenosis rates were calculated and are shown in Table 1. There was no significant difference between venous diameter or stenosis rate based on sex (Supplementary Table 1). The length of the animal thrombus was 30.7 ± 2.8 mm. In this process, we sequentially injected 1 ml of thrombin and fibrinogen under direct vision and occluded the blood flow with vascular clamps for 10 min. Routine blood tests, blood biochemistry, and coagulation function were performed before the operation and 7, 30, 60, and 90 days after the operation (Fig. 2). Increased white blood cell, neutrophil, and monocyte counts were present 7 days after surgery compared to 0 days, but the symptoms of haemogram infection had been controlled 30 days after surgery. There was no evident abdominal wound bleeding or signs of wound infection during postoperative anti-infection treatment.

**Table 1:** The underlying condition and intraoperative data of dogs and pigs

Mean*:t* SD or N(¾)	Canine (*n* = 6)	Plg(*n* = 3)	*P* value^*^
Sex(M)	3(50%)	2 (66.7%)	0.64
Animal weight (kg)	27.8 ± 4.6	32.l ± 3.4	0.20
Duration of operation (min)	74.3 ± 14.7	143.7 ± 33.1	< 0.05
Venous perimeter (mm)	32.0 ± 3.3	31.7 ± 1.5	0.88
Venous diameter (mm)	10.2 ± I.I	10.1* ±* 0.5	0.87
Remeasured venous perimeter (mm)	12.7 ± 2.4	13.7 ± 1.5	0.54
Remeasured venous diameter (mm)	4.1 ± 0.8	3.6 ± 0.8	0.57
Thrombosis length (mm)	30.7 ± 2.8	27.3 ± 2.3	0.2
Stenosls rate (%)	83.6 ± 6.4	86.2* ± *5.7	0.56

*M* male, *N* number, *SD* standard deviation, *kg* kilogram, *min* minute, *mm* millimeter

^*^P value, comparison vs. canine group

**Fig. 3:**
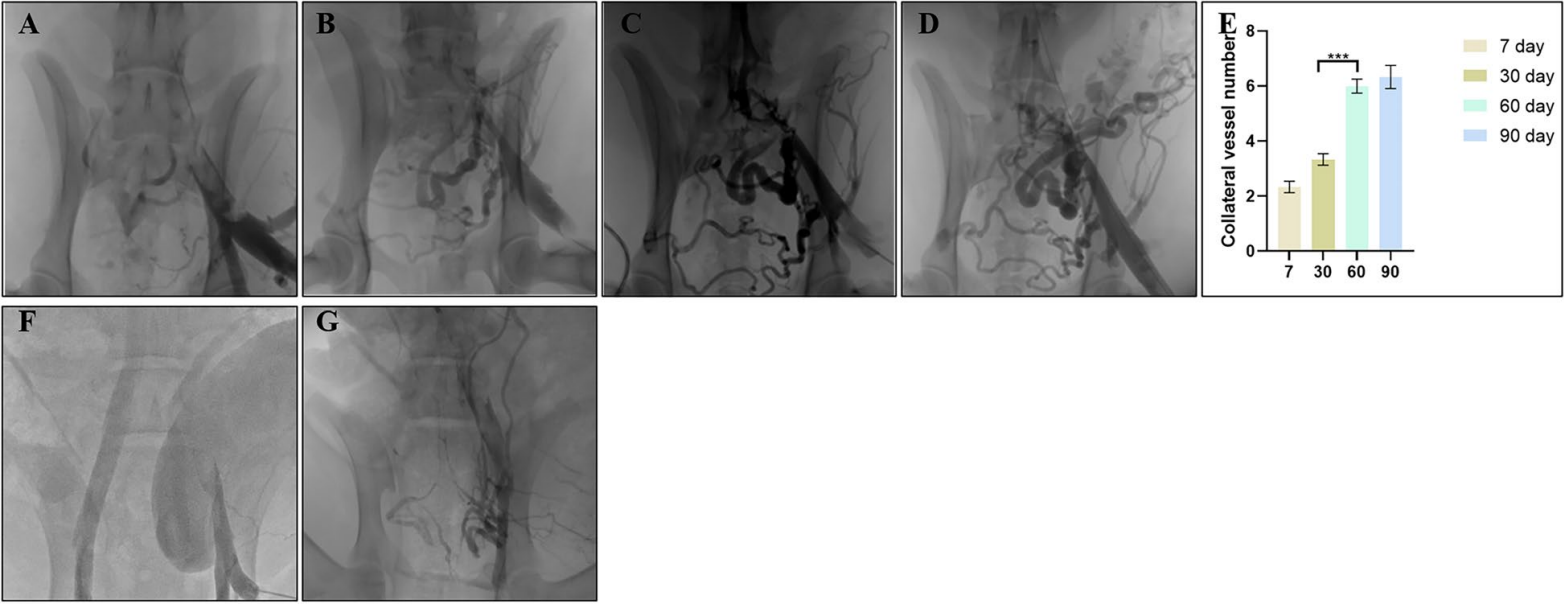
Venous angiography. **A**-**D** 90-day follow-up venous angiography of canines. A, Days 7; B, Days 30; C, Days 60; D, Days 90. **E** Numbers of collateral vessels in canines. *** *P* < 0.001 60 days vs. 30 days. **F**, **G** Thirty-day follow-up venous angiography of pigs. E, Days 7; F, Days 30

Clinically, patients with chronic iliofemoral vein thrombosis have angiographic manifestations of vein occlusion or smaller caliber, with significant collateral circulation and distal dilation. All animals underwent antegrade venography of the left iliac veins at follow-up time points to observe the formation of thrombi and the restriction of blood flow. As shown in Fig. 3, 7 days after modelling, no contrast material passed through the left common iliac vein in any of the animals. A small amount of collateral circulation was observed, the thrombus segment was visible, and the distal vessel was distended. At 30 days, angiography of the thrombus showed discontinuous filling defects or narrowing of the lumen (Fig. 3B). The possible cause was thrombolysis and thrombus organization because of postoperative anticoagulant drugs, which attached to the vein wall and caused blood flow restriction. Collateral compensation was significantly increased at 60 days compared to 30 days after modelling, and distal limb blood flow returned to the heart from the collaterals (Fig. 3C, E). At 90 days, the collateral circulation gradually stabilized, the collateral vessels became thicker, and the blood flow was dominated by several major collateral branches (Fig. 3D).

**Fig. 4:**
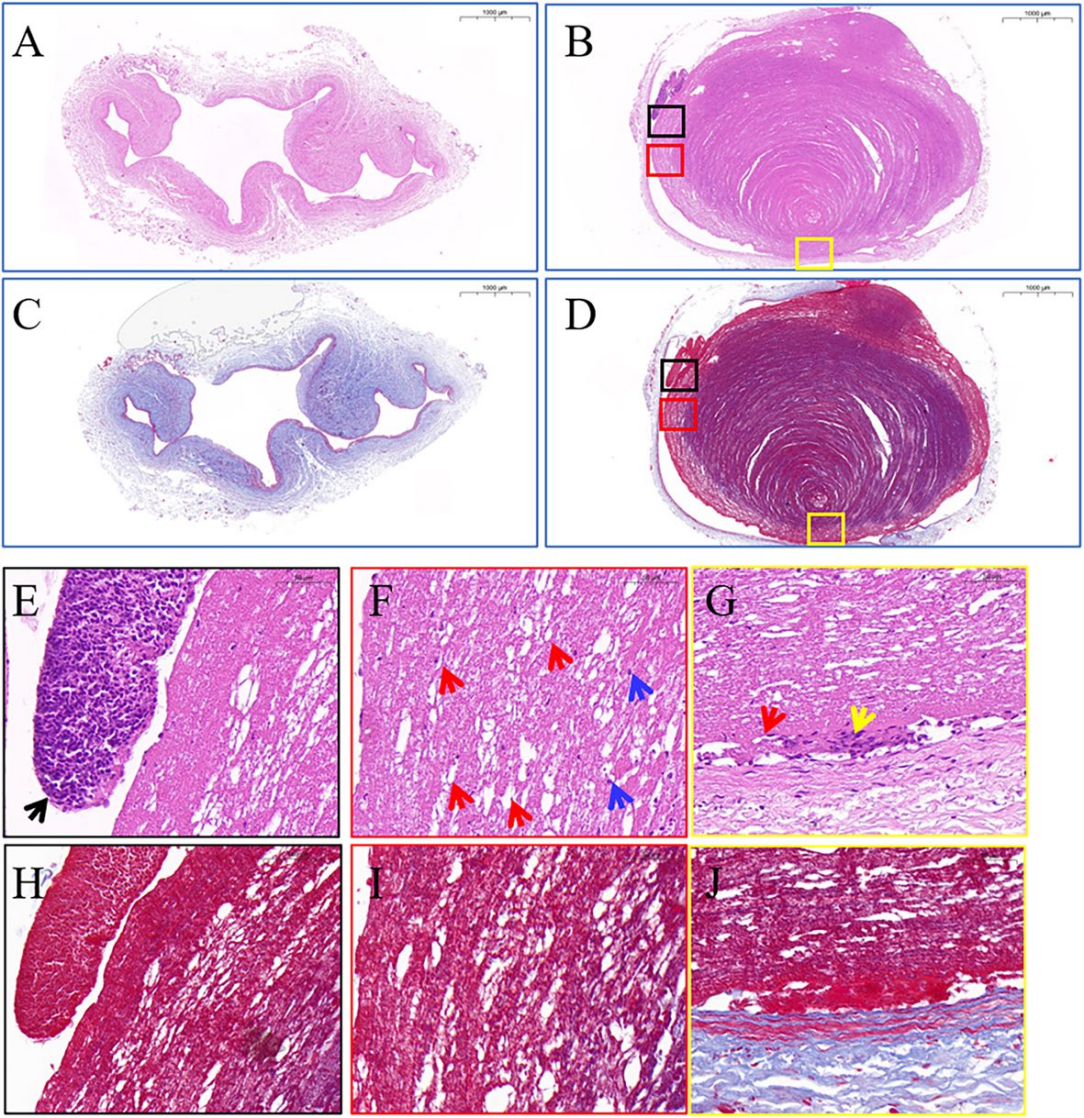
Images of HE and Masson staining of bilateral iliac veins 90 days after modelling in dogs. **A** HE staining of the right iliac veins. **B** HE staining of left iliac venous thrombosis. **C** Masson staining of the right iliac veins. **D** Masson staining of left iliac venous thrombosis. Scale bars: 1000 μm. **E**–**G** Magnified HE staining of left iliac venous thrombosis. Black arrows, attached white blood cells; red arrows, capillaries; blue arrows, degenerated white blood cells; yellow arrows, fibroblasts. Scale bars: 50 μm. (H-J) Masson staining of left iliac venous thrombosis. Scale bars: 50 μm

Histological staining of the bilateral iliac veins was performed on Day 90 of thrombosis. The lumen structure of the contralateral iliac vein was clear and collapsed in an irregular shape, and there was no thrombus in the lumen (Fig. 4A). In Fig. 4C, Masson staining is visible in the venous wall film of approxiamately 3–5 continuous layers of deep purple elastin, lining layer with a thin layer of elastin. In the thrombosis model group, as shown in Fig. 4B, the vascular lumen was dilated and filled with thrombi, and one side was close to the vessel wall. In the thrombus, the fibrin meshwork and the platelet trabecula met each other, showing concentric circles. There were varying numbers of degenerated leukocytes attached to the fibrin meshwork (indicated by the blue arrow), and more leukocytes were attached locally on one side of the thrombus and aggregated into masses (black box) in Fig. 4E, F. However, the peripheral part of the thrombus and the attachment had begun to organize, and some capillaries and a few fibroblasts were visible (red and yellow box) in Fig. 4G. Masson staining showed a tremendous amount of dyed purple blood clots fibrin in Fig. 4D. Magnification illustrates attached white blood cells as shown by black arrows, capillaries as shown by red arrows, degenerated white blood cells inside the thrombus as shown by blue arrows, and a small number of proliferating fibroblasts as shown by yellow arrows.

### The porcine model of chronic deep venous thrombosis

For some reason, three Bama miniature pigs died on Days 29, 33, and 58 after venous thrombosis formation. The animal monitors did not see any noticeable abnormalities during daily inspections until the animals were found to die suddenly, without any signs before death, and the surgical sites were incised, sutured tightly and well healed. In Table 1, there was no significant difference between canines and pigs except for the duration of surgery. The time of thrombus modelling in pigs was much longer than that in dogs due to the complex lower abdominal and pelvic anatomy of pigs (Supplementary Fig. 1). The inferior vena cava and bilateral iliac veins of pigs were in the fat-filled retroperitoneal tissues, affecting vascular separation and exposure (Supplementary Fig. 1A). The diameter and length of the left iliac veins were similar, and the surgical procedure was consistent with that in dogs. Postoperative haematologic testing revealed decreased albumin levels in the pigs at 30 days compared with those at 0 days (Supplementary Fig. 2H). Furthermore, in Fig. 3G, at 30 days, only a few and tiny collateral branches appeared. Autopsy reports of three animals suggested thoracic infection, tissue bruising and pulmonary haemorrhage as the cause of death (Supplementary Fig. 3).

## Discussion

DVT is a common disease [16]. Establishing animal models that conform to the characteristics of human thrombosis is a necessary method to reveal the pathogenic mechanism and to research drugs and devices that can improve treatment strategies. Small animal models, especially rodents, are the most commonly used research subjects. However, due to their size differences, small animal models cannot be used for translational research of various medical devices, which limits the development of new interventional or open surgery techniques. In approximately half of large animal thrombosis studies, pig have been chosen as the large animal model for venous thrombosis research because pigs have a similar vascular anatomy and size to humans, allowing researchers to test therapeutic drugs, devices and surgical procedures, to assess DVT imaging scans and to study DVT pathophysiology [17]. However, a load of pelvic and lower limb blood reflux due to inferior vena cava or iliofemoral vein thrombosis makes pressure-sensitive pigs susceptible to death, with a mortality rate of up to 33% [18]. Compared with other large experimental animals, such as pigs or sheep, dogs are more compliant and easier to handle during anaesthesia and follow-up, while they also have stronger resistance to infection and higher survival rates. More importantly, in the chronic phase of human DVT, it is emphasized to increase the amount of activity to avoid the spread and recurrence of thrombosis caused by blood stasis [19]. The active nature of dogs is more consistent with the development of chronic thrombosis in the human body. The diameters of veins used in DVT models varied from 6.3 mm to 14 mm in previous studies [17]. In this study, the diameter of the left common iliac vein in Labrador dogs was 8.6–11 mm, which was close to the diameter of the human iliac vein when studying interventional medical devices.

The most common method of modelling venous thrombosis is the venous stasis model, in which blood flow is restricted or temporarily blocked by ligation of varying degrees of stenosis, balloon blockade, or cone stenting [20], and local thrombus formation is induced by exogenous injection of thrombin. There is also significant thrombus formation in the lumen by stimulation of the vascular endothelium by anodal direct current [21]. Notably, in models subjected to the intraluminal technique, flow-blocking using both ends of the balloon and catheter administration of thrombin to promote thrombus formation, the balloon block time was maintained for at least 1 h, regardless of vessel diameter (jugular, iliac, inferior vena cava) [22]. The time needed was longer when the electrical stimulation method was used [21]. In this experiment, the innovative use of both thrombin and fibrinogen resulted in the formation of a visibly solid thrombus within approximately 5–7 min of the intraoperative blockade, greatly reducing the time of the modelling procedure and mitigating the risk of anaesthesia in the animals.

The canine model of chronic thrombosis in this study recapitulates the features of post-thrombotic syndrome in humans. In addition, this study is the first to use angiography to observe thrombosis for up to 90 days, providing a model that is consistent with chronic thrombosis progression. Furthermore, the measurable stenosis rate of venous obstruction can be divided into different groups to determine the timing of intervention. This model can be used to study the biological safety and efficacy of anticoagulant drugs, thrombectomy, thrombolytic devices, and various new venous stents for venous obstruction. We compared the ability of pigs and dogs to tolerate long-term chronic thrombosis and showed that pigs died suddenly due to thoracic infection, tissue bruising, and pulmonary haemorrhage. Possible triggers were prolonged blood flow obstruction due to massive thrombosis of the common iliac vein, including restricted internal iliac vein return, poor collateral compensation, and severe pelvic stasis, which induced gastrointestinal stress and led to malnutrition and infection. In addition, pigs may have poor sensitivity to anticoagulant drugs and higher susceptibility to bleeding, which also influences the successful establishment of the model. Moreover, dogs have superior health, obedience, and proneness to activity than pigs, which may be the reason why establishing the canine model is more successful.

### Supplementary Information

**Additional file 1: Supplementary Table 1.** The underlying condition and intraoperative data of dogs. SD, standard deviation; kg, kilogram; min, minute; mm, millimetre. * *P *value, comparison vs. male group.

**Additional file 2: Supplementary Fig. 1.** Induced venous thrombosis in the left iliac veins of pigs. (A) Separated bilateral iliac veins. (B, C) Measured iliac vein circumference. (D) Diameter of the ligation rod. (E) Ligation of the rod and left iliac vein. (F, G) Remeasured iliac vein circumference. (H) Thrombosis length. (I) Injection with thrombin and fibrinogen.

**Additional file 3: Supplementary Fig. 2.** Haematological testing of pigs. * *P*<0.05, ** *P*<0.01, *** *P*<0.001, all groups vs. 0 day.

**Additional file 4: Supplementary Fig. 3.** Autopsy reports of three Bama miniature pigs. (A, B) 29 days. (C, D) 33 days. (E, F) 58 days.

## Data Availability

All data and materials in this research will be freely available to any scientist wishing to use them for non-commercial purposes, without breaching participant confidentiality.

## References

[1] Deep Duffett L, Thrombosis Venous (2022). Deep Venous Thrombosis. Ann Intern Med..

[2] Diaz JA, Saha P, Cooley B (2019). Choosing a mouse model of venous thrombosis: a consensus assessment of utility and application. J Thromb Haemost.

[3] Hu N, Kong LS, Chen H (2015). Autophagy protein 5 enhances the function of rat EPCs and promotes EPCs homing and thrombus recanalization via activating AKT. Thromb Res.

[4] Kong L, Hu N, Du X (2016). Upregulation of miR-483–3p contributes to endothelial progenitor cells dysfunction in deep vein thrombosis patients via SRF. J Transl Med..

[5] Baldwin MJ, Moore HM, Rudarakanchana N, Gohel M, Davies AH (2013). Post-thrombotic syndrome: a clinical review. J Thromb Haemost.

[6] Levi M, Dörffle-Melly J, Johnson GJ, Drouet L, Badimon L (2001). Usefulness and limitations of animal models of venous thrombosis. Thromb Haemost.

[7] Gromadziński L, Paukszto Ł, Skowrońska A, et al. Transcriptomic Profiling of Femoral Veins in Deep Vein Thrombosis in a Porcine Model. Cells. 2021;10(7). 10.3390/cells10071576. 10.3390/cells10071576 PMC8304794 34206566

[8] Schwein A, Magnus L, Markovits J (2022). Endovascular Porcine Model of Iliocaval Venous Thrombosis. Eur J Vasc Endovasc Surg.

[9] Geier B, Barbera L, Muth-Werthmann D (2005). Ultrasound elastography for the age determination of venous thrombi. Evaluation in an animal model of venous thrombosis. Thromb Haemost..

[10] Kang C, Bonneau M, Brouland JP, Bal dit Sollier C, Drouet L (2003). In vivo pig models of venous thrombosis mimicking human disease. Thromb Haemost..

[11] Shi WY, Hu LY, Wu S, Liu CJ, Gu JP (2015). Two swine models of iliac vein occlusion: Which form most contributes to venous thrombosis?. Thromb Res.

[12] Becker EM, Perzborn E, Klipp A (2012). Effects of rivaroxaban, acetylsalicylic acid and clopidogrel as monotherapy and in combination in a porcine model of stent thrombosis. J Thromb Haemost.

[13] Greiten LE, McKellar SH, Rysavy J, Schaff HV (2014). Effectiveness of rivaroxaban for thromboprophylaxis of prosthetic heart valves in a porcine heterotopic valve model. Eur J Cardiothorac Surg.

[14] Hafner PM, Mackin AJ, Wills RW, Brooks MB, Thomason JM (2022). Anticoagulant effects of rivaroxaban, prednisone, alone and in combination, in healthy dogs. J Vet Intern Med.

[15] Weinz C, Schwarz T, Kubitza D, Mueck W, Lang D (2009). Metabolism and excretion of rivaroxaban, an oral, direct factor Xa inhibitor, in rats, dogs, and humans. Drug Metab Dispos.

[16] Di Nisio M, van Es N, Büller HR (2016). Deep vein thrombosis and pulmonary embolism. Lancet.

[17] Schwein A, Magnus L, Chakfé N, Bismuth J (2020). Critical Review of Large Animal Models for Central Deep Venous Thrombosis. Eur J Vasc Endovasc Surg.

[18] Hosaka J, Roy S, Kvernebo K, Enge I, Laerum F (1996). Induced thrombosis in the pig inferior vena cava: a model of deep venous thrombosis. J Vasc Interv Radiol May-Jun.

[19] Kyrle PA, Eichinger S (2005). Deep vein thrombosis. Lancet..

[20] Lin PH, Chen C, Surowiec SM, Conklin B, Bush RL, Lumsden AB (2001). Evaluation of thrombolysis in a porcine model of chronic deep venous thrombosis: an endovascular model. J Vasc Surg.

[21] Aruva MR, Daviau J, Sharma SS, Thakur ML (2006). Imaging thromboembolism with fibrin-avid 99mTc-peptide: evaluation in swine. J Nucl Med.

[22] Goudot G, Khider L, Del Giudice C (2020). Non-invasive recanalization of deep venous thrombosis by high frequency ultrasound in a swine model with follow-up. J Thromb Haemost.

