# Supplementary figures and images for: Comparison between canine and porcine models of chronic deep venous thrombosis
Source: Thromb J. 2023 Dec 6;21:121. doi: 10.1186/s12959-023-00565-5 (PMC10702023; doi:10.1186/s12959-023-00565-5)

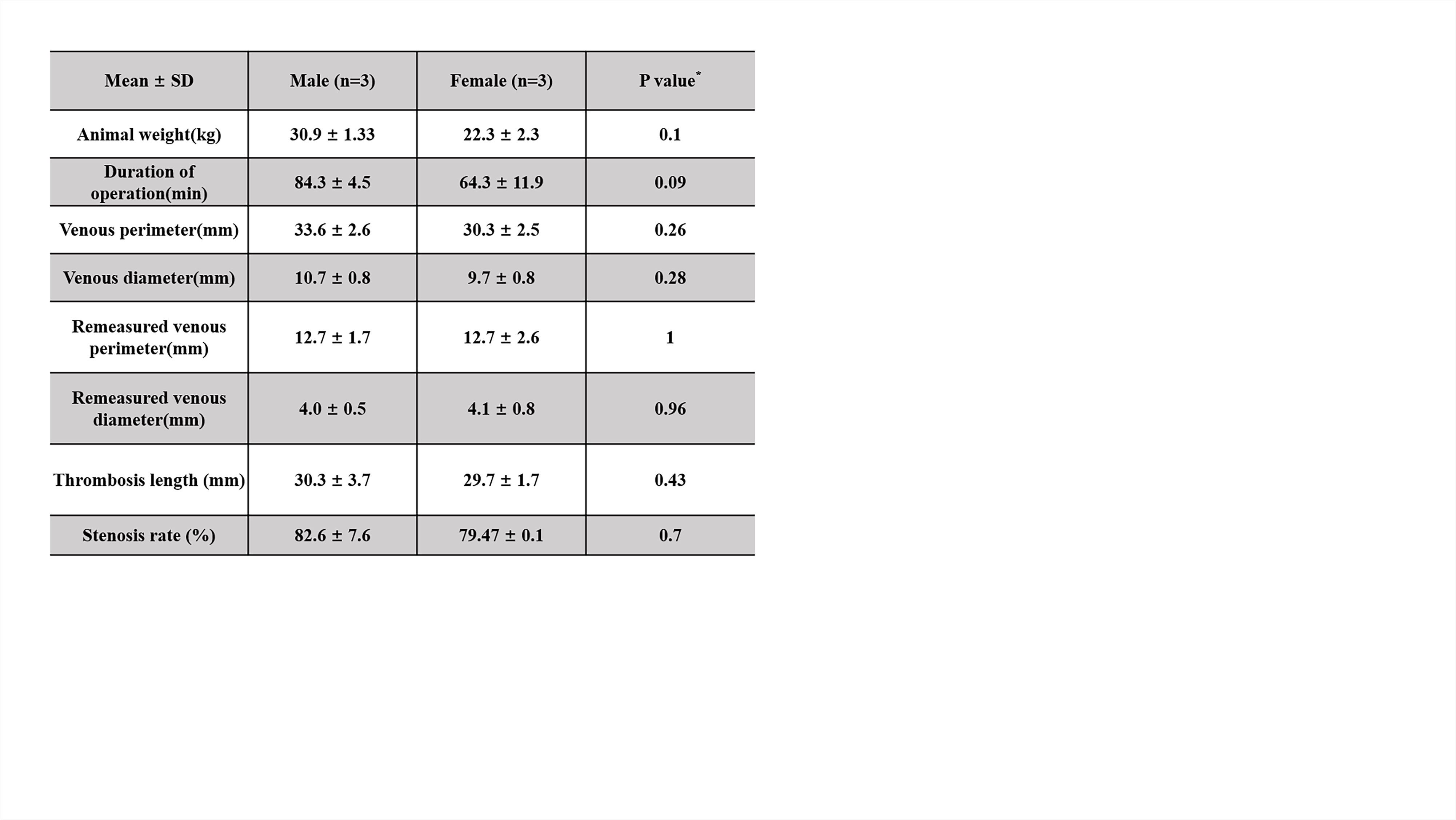

Supplement: Supplementary file 1 — Additional file 1: Supplementary Table 1. The underlying condition and intraoperative data of dogs. SD, standard deviation; kg, kilogram; min, minute; mm, millimetre. * P value, comparison vs. male group. [file 12959_2023_565_MOESM1_ESM.tif]

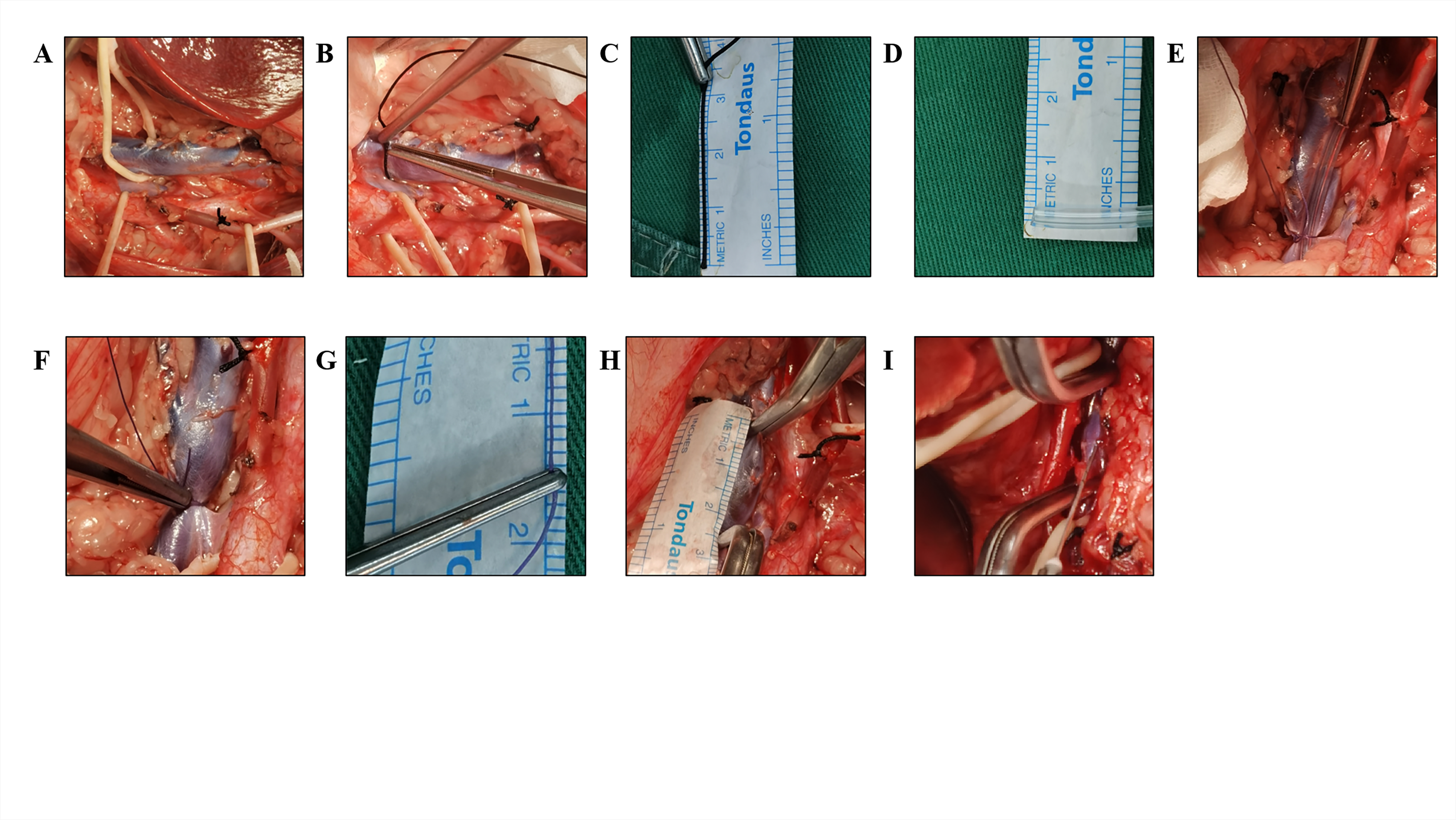

Supplement: Supplementary file 2 — Additional file 2: Supplementary Fig. 1. Induced venous thrombosis in the left iliac veins of pigs. (A) Separated bilateral iliac veins. (B, C) Measured iliac vein circumference. (D) Diameter of the ligation rod. (E) Ligation of the rod and left iliac vein. (F, G) Remeasured iliac vein circumference. (H) Thrombosis length. (I) Injection with thrombin and fibrinogen. [file 12959_2023_565_MOESM2_ESM.tif]

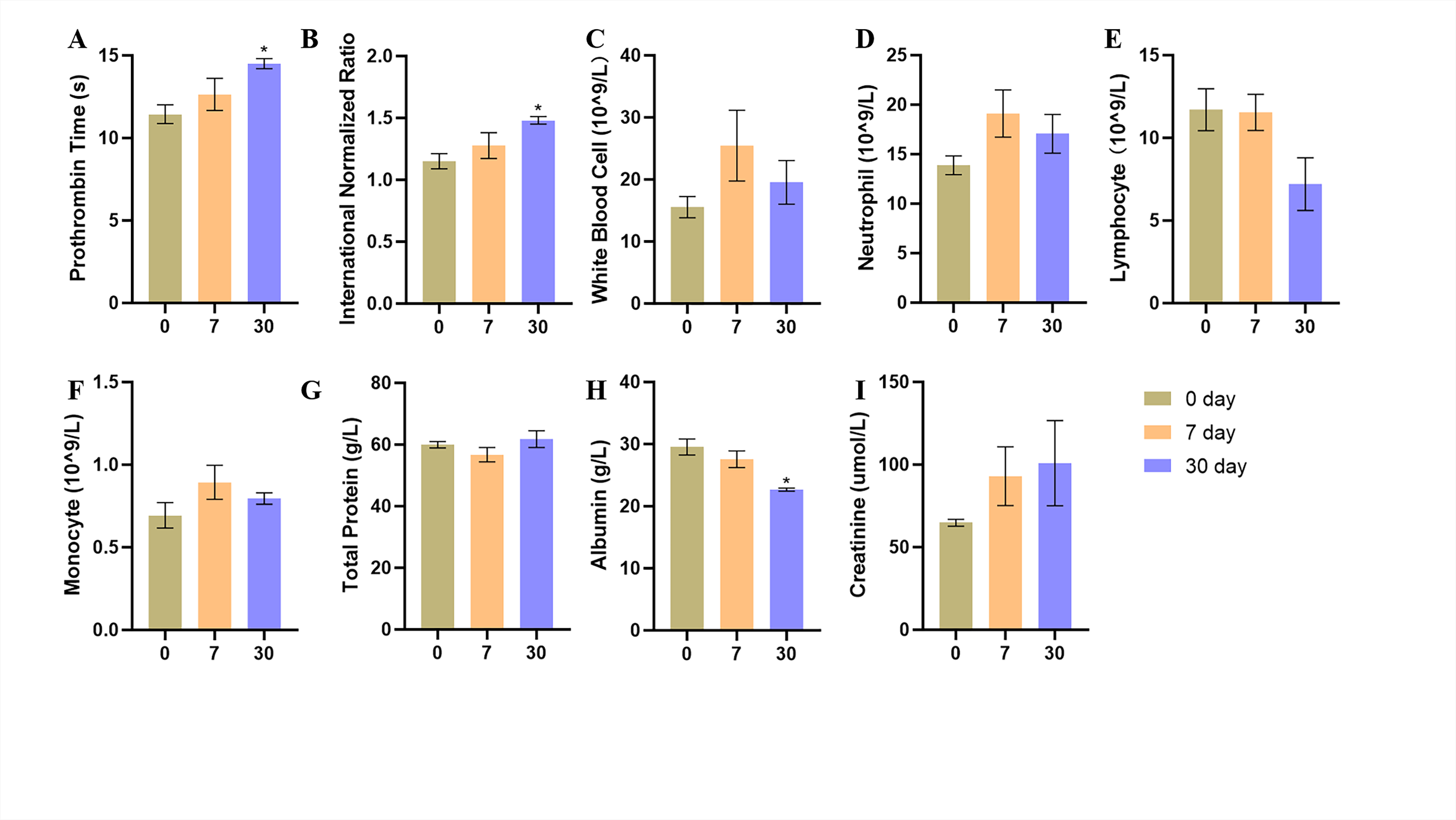

Supplement: Supplementary file 3 — Additional file 3: Supplementary Fig. 2. Haematological testing of pigs. * P<0.05, ** P<0.01, *** P<0.001, all groups vs. 0 day. [file 12959_2023_565_MOESM3_ESM.tif]

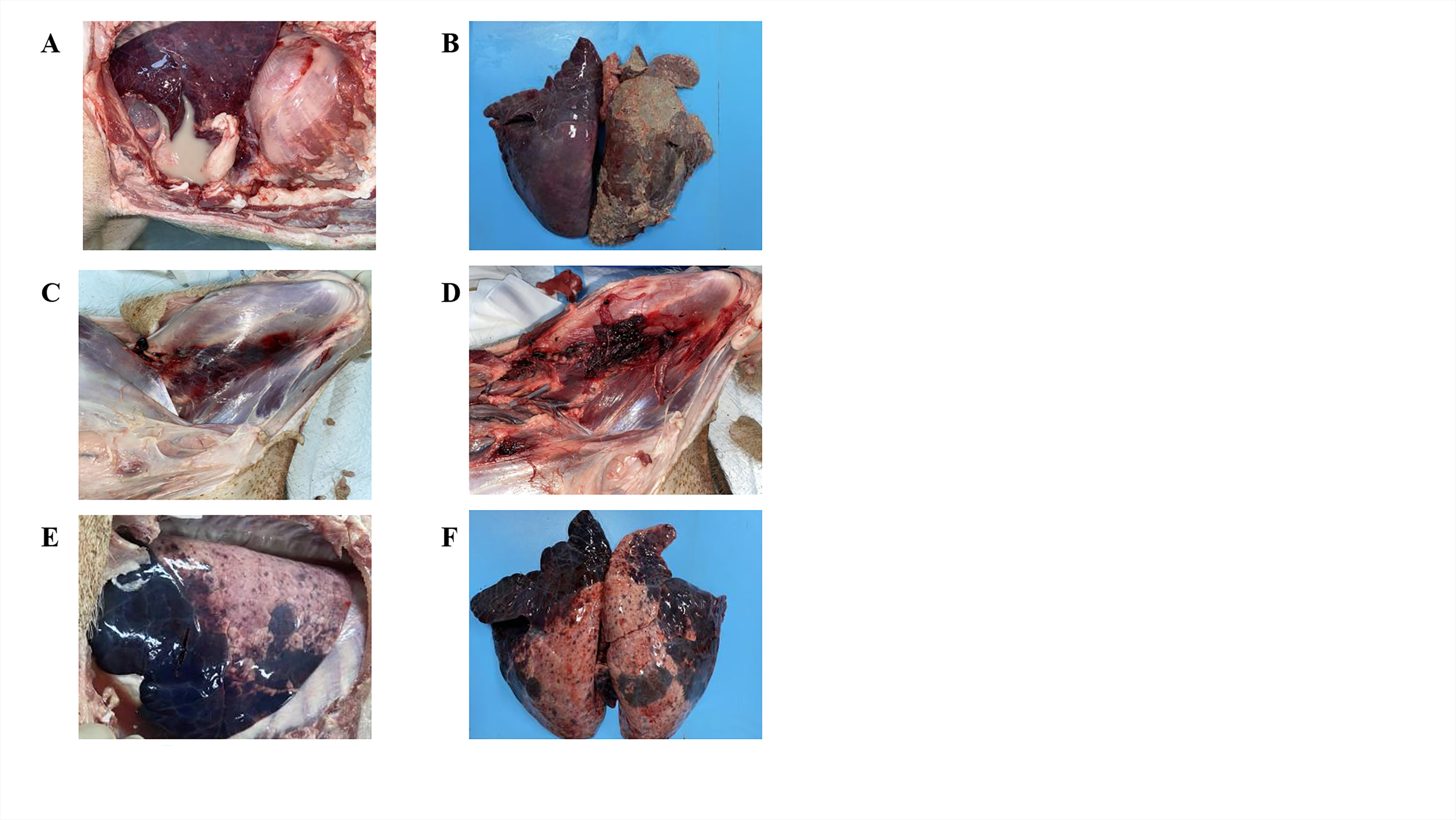

Supplement: Supplementary file 4 — Additional file 4: Supplementary Fig. 3. Autopsy reports of three Bama miniature pigs. (A, B) 29 days. (C, D) 33 days. (E, F) 58 days. [file 12959_2023_565_MOESM4_ESM.tif]
